# Experimental Study on the Thermoplastic Dripping and Flame Spread Behaviors of Energized Electrical Wire under Reduced Atmospheric Pressure

**DOI:** 10.3390/polym13030346

**Published:** 2021-01-22

**Authors:** Hao He, Qixing Zhang, Long Shi, Haihang Li, Dongmei Huang, Yongming Zhang

**Affiliations:** 1 College of Quality and Safety Engineering, China Jiliang University, Hangzhou 310018, China; hehao@cjlu.edu.cn (H.H.); lihaihang@cjlu.edu.cn (H.L.); dmhuang@cjlu.edu.cn (D.H.); 2 State Key Laboratory of Fire Science, University of Science and Technology of China, Hefei 230026, China; zhangym@ustc.edu.cn; 3 Civil and Infrastructure Engineering Discipline, School of Engineering, RMIT University, Melbourne 3004, Australia; shilong@mail.ustc.edu.cn

**Keywords:** dripping behavior, molten thermoplastic, reduced pressure, energized wires, flame extinction

## Abstract

Flame spread over wire surface is different from other solid fires as it is usually accompanied by melting and dripping processes. Although the related behaviors at reduced pressure (20–100 kPa) are significant to those fire risk evaluations, very few studies have been undertaken on this matter. Therefore, the thermoplastic dripping and flame spread behaviors of energized polyethylene insulated copper wires were investigated experimentally at reduced pressure. It was known from experimental results that the dripping frequency increases, showing a relatively smooth (linear) and rapid (power) increasing trends under high and low electrical currents, respectively. A short-period flame disappearance was observed during the dripping process, which is unique for the energized wire at reduced pressure. The bright flame can disappear for several seconds and then show again after the dripping. While at 20 kPa or lower, the wire flame would turn to a completed extinguishment after the dripping. A critical dripping point was proposed to show the minimal required electrical current to sustain the flame spearing. The critical current changes smoothly during 100–80 kPa and decreases rapidly at 80–60 kPa. Additionally, the dripping phenomenon can stop or delay the flame spread, partly because of the short-term flame disappearance.

## 1. Introduction

Thermoplastic polymer material, such as polyethylene and acrylonitrile butadiene styrene plastic, have attracted considerable attention in research and industry [1,2]. However, the dripping of molten thermoplastic can greatly increase the level and risk of a fire as it could easily result in the ignition of surrounding combustibles and a quick expansion of the burning front and area. Especially for those wires under overcurrent, the flame spreads relatively faster along with the energized wire under the molten and dripping matrix [3]. As those wires are compulsory in our daily lives, it is critical to understand its fire spread and dripping behaviors under fire conditions that enable to take actions to present the fire spread and reduce the relevant fire risks.

A large number of studies have been undertaken previously on addressing the fire behaviors of wires [4]. The influencing factors of the relevant fire spreading considered in these studies including inclination angles [5], AC electrical fields [6], oxygen concentration [7], external radiation intensity [8], ambient pressure [9,10,11], and gravity [12,13,14,15]. Besides these influencing factors, their ignition behaviors were investigated under various scenarios, such as short-term overcurrent [16,17], external heat flux [18], arc formation [19,20], subatmospheric [21,22], and microgravity [23,24]. A few studies have also been carried out on investigating the thermoplastic properties of those melt drips [25,26,27,28]. As dripping is a common phenomenon for wire fires, the effects of melting and dripping behaviors on flame spread and the follow-up extinction were also addressed [3,5,13,29,30].

Ignition, flame spreading and the accompanied dripping phenomenon of electrical wires under a standard atmospheric pressure were investigated in the previous studies experimentally [31,32,33]. The formation of drips and the dripping frequency, as important outputs to determine the fire risk, were investigated. A model was developed to address the relationship between the molten mass loss and the volume change to predict the time period and frequency of the dripping phenomenon under various fire conditions.

Although many studies have focused on the ignition and flame spread of an electrical wire, very few studies were reported in the literature that addressed the influences of dripping behaviors on flame spread under reduced pressure, for the wire used in aerospace and the Lhasa city with altitude of 3650 m; 64 kPa. Under reduced pressure, the decreased oxygen concentration and surrounding pressure could largely affect the burning and dripping behaviors. As they are rarely reported previously, the related dripping behaviors and fire risk are still not known that has largely hampered the related fire risk evaluation under this special scenario.

Therefore, through this study, the influences of reduced atmospheric pressure on the molting and dripping behaviors of those energized wires were investigated experimentally. The research outcomes provide a practical guideline on evaluating the fire risk and promoting fire suppression.

## 2. Experimental Methodology

**Figure 1:**
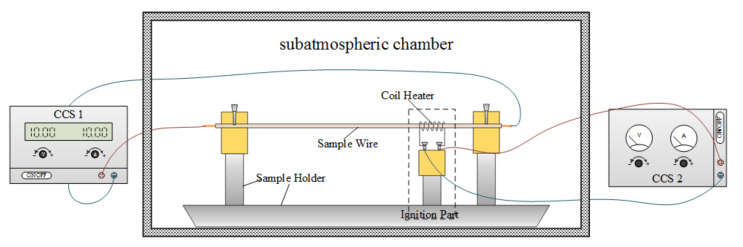
Schematic of the experimental apparatus.

As shown in Figure 1, the experimental apparatus consists of three parts, namely a wire sample holder, two constant current sources (CCSs) and an ignition device. The sample holder was used to keep the wire sample straight and tight during the whole process. The coil heater, which was made of Nicochrome, could function as an igniter when energized. The CCSs were used to adjust the current of the sample wire and precisely control the current imposed on the coil heater. To enable a reduced atmospheric pressure, the experimental apparatus was placed in a customized-designed subatmospheric chamber. It can achieve a low pressure of 7.5 kPa with an accuracy of 0.1 kPa. The changing rate of the internal pressure can be less than 0.1 kPa/min, and several primary tests have confirmed its airtight performance. The experimental apparatus provides an ideal condition for reduced-scale wire fire tests.

A type of copper wire was used in this study. Two CCS were placed outside the chamber to control the current through the wires. After the wire sample was placed on the holder, the front door of the chamber was closed to enable a sealing environment. Then the target pressure was set and the vacuum pump then started working on pulling out the air. When the internal pressure was at the set value, the pump stopped and 2–3 min was needed to enable a stable pressure. After that, CCS 1 was then turned on to energize the sample wire, and about 2 min were required before the wire temperature increased from the ambient temperature to the equilibrium temperature. Then CCS 2 was turned on to supply the coil heater for ignition. When the flame passed through the ignition region, CCS 2 was then turned off immediately to avoid extra heat radiation. CCS 1 was kept working until the wire burned out or the flame extinction.

During the whole test, a camera was placed outside the front door of the chamber, through an observation window made of heat-resistant glass, to record the whole burning process. The flame spread and dripping phenomenon were then analyzed through the image processing. To minimize the influence of the external environment, all tests were conducted under an ambient temperature within 20–25 °C and relative humidity of 70–80%. At least five repeated tests were conducted to enable good repeatability of the experiments. The experimental conditions are listed in Table 1. More tests relating to the critical dripping point were conducted to obtain more accurate result.

## 3. Results and Discussion

### 3.1. Flame Spread Rate (FSR)

**Figure 2:**
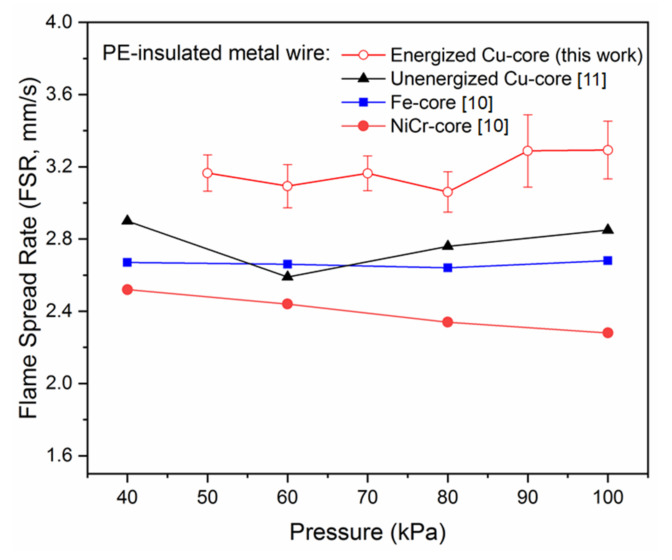
Effects of pressure on the flame spread rate (FSR) along the polyethylene- insulated metal wires.

Figure 2 presents a comparison between this study and previous studies, including NiCr-core and Fe-Core wires tested by Nakamura [10] and the unenergized Cu-core wire reported by Hu [11]. It can be seen that FSR is generally higher for the Cu-core wires than those of Fe-core and NiCr-core wires, especially for those energized wires. However, the variation of reduced pressure on FSR is not significant, as the effect on NiCr harness is the largest with the maximum variation ratio of less than 10%. In another word, FSR is less affected under a reduced pressure of 40–100 kPa.

**Figure 3:**
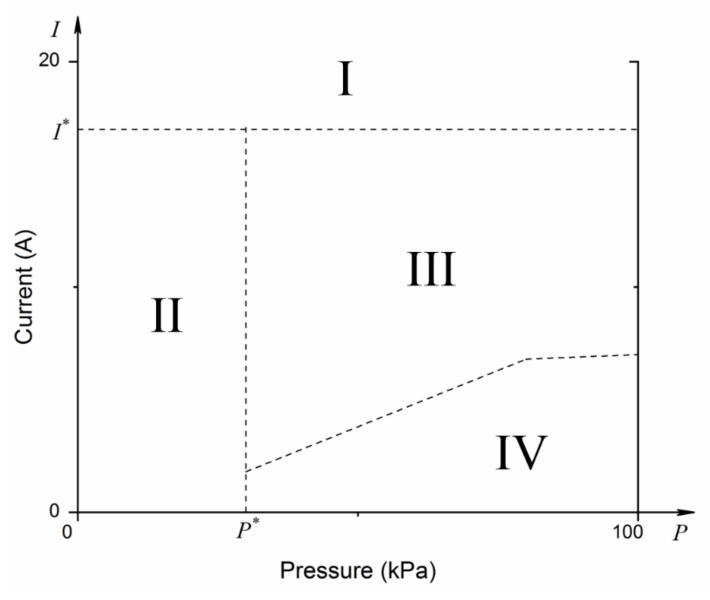
The four sections for energized wire at reduced pressure.

Based on the experimental results, the burning and dripping phenomena for the energized wire at reduced pressure could be divided into four sections, as shown in Figure 3. For Section I namely the overheated region, the metal core is overheated due to the excessive current. It leads to a short-term but rapid volatilization of the insulating polyethylene which fuses or worsens the metal core. The flame spread cannot be maintained under the scenario. There exist a critical current $I^{*}$ for the demarcation line, and $I^{*}/I_{0}>20$ can be used in this study. For Section II, namely the flame extinguish region, as the pressure is too low (lower than the critical pressure, $P^{*}$) the flame cannot maintain along the wire, even under the situation the wire cannot be ignited. Section III is the continued dripping region, where flame spreads along the wire accompanied by the molten dripping behavior. For Section IV, where the overload current is relatively small, both of the flame spread and the flame morphology are steady, and no dripping phenomenon was observed. Between Sections III and IV, there is a demarcation line, which is associated to the overload current and the reduced pressure.

### 3.2. Dripping Frequency

The melt dripping frequency could be higher at a reduced pressure under the weakened convection and decreased pyrolysis rate. As in the previous study [31], dripping time is defined as the adjacent time interval for the drops, while the dripping frequency (f) is the reciprocal of the dripping time. The dripping frequency can be also determined by the ratio between the accumulated mass of molten insulation and the average mass of one drop, namely $f={\dot{m}}_{mlt}/\rho_{p}V_{lim}$. The mass of molten insulation (${\dot{m}}_{mlt}$) represents the relationship between the mass-loss of insulation (${\dot{m}}_{p}$) and the insulation pyrolysis rate (${\dot{m}}_{f}$) [31]:

(1) $${\dot{m}}_{mlt}={\dot{m}}_{p}-{\dot{m}}_{f},\;{\dot{m}}_{p}=\rho_{p}A_{p}v_{f}$$

Here, the FSR ($v_{f}$) is not sensitive to the ambient pressure (*P*) as shown in Figure 1. Additionally, the burning rate ${\dot{m}}_{f}$ is proportional to the overall reaction rate [34] ${{\dot{m}}^{\prime \prime \prime }}_{f}\;=\rho_{g}A_{0}Y_{F}^{n}Y_{O_{2}}^{n}\exp(-E/RT)$.

**Figure 4:**
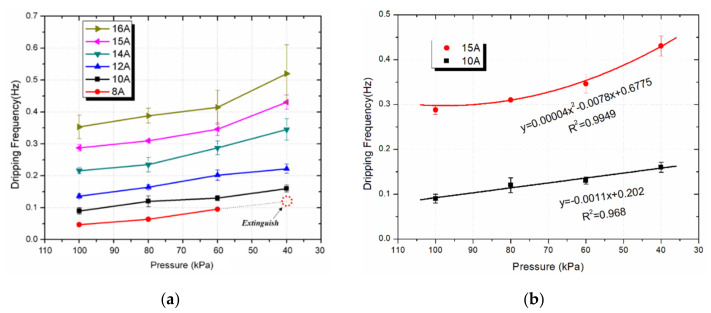
Dripping frequency at various pressures for the energized wire: (**a**) wire under various currents; (**b**) the different growth rates for wire under specific currents.

The Arrhenius formula shows that the burning rate is reduced as the pressure decreases, therefore the dripping frequency has a negative correlation with the ambient pressure. Figure 4a shows the relationship between the dripping frequency and the ambient pressure for the energized wire. Our hypothesis regarding the influences of the pressure on the dripping frequency has also been confirmed through the experimental results in Figure 4. The curves show the same trend with those under the standard atmospheric pressure that the dripping frequency keeps increasing under reduced pressure. The increasing trends are similar even the wires are under different electrical currents.

The effects of the electrical current on the dripping frequency are quite obvious. It can be seen that the electric current can obviously increase the dripping frequency, as shown in Figure 4a. The electrical current could also determine the burning process of the wire. For example, no sustained flame was observed when the energized wire was under 8 A electrical current, while the flame spread can be seen for the wire under an electrical current of 10 A or above.

It was known that the dripping frequency goes higher under lower atmospheric pressure, but they showed different growth rates under various electrical currents. For example, the curves for I = 10 A and I = 15 A are compared separately, as shown in Figure 4b. Under a relatively lower current (I = 10 A), the dripping frequency increases almost linearly, while it shows a power function under a higher current (I = 15 A). The effect of the current is much more obvious under a bigger current.

During the burning process, part of the burning combustibles sometimes separates from the wire, which results in a sudden mass loss and insufficient heat transfer from the flame to molted PE. A higher dripping frequency can lead to a relatively bigger mass loss. There needs a minimal amount of heat to sustain the wire fire. Once it is below the limit, the flame spreading process could be hardly maintained. After dripping occurs, the bright flame could disappear for a short period of time, or even ultimately extinguished.

### 3.3. Flame Extinction Induced by Molten Dripping Behavior

**Figure 5:**
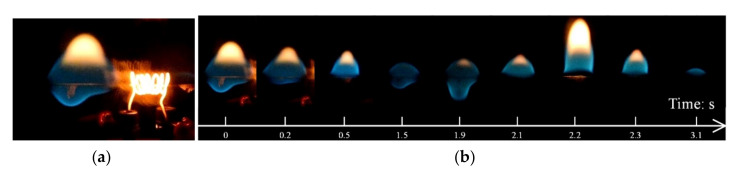
The flame morphology along with the time at 20.0 kPa: (**a**) dripping occurred just behind the ignition and (**b**) the dripping and flame extinction process.

Under the situation when the ambient pressure kept decreasing, wire fire turned to being difficult to be sustained (Section 2 in Figure 3). For example, at 20.0 kPa, the sample wires were hard to be ignited due to the seriously low oxygen mass-concentration. Only under very extreme current such as I = 15 A, the wire could be ignited. As shown in Figure 5a, the flame happened in the heating zone so that a bright wire could be observed. The flame then moved to the left along with the horizontal wire. Dripping occurred immediately when the flame passed through the heating zone. In the blue flame area, melt droplets were formed and began to fall.

The whole dripping process is shown in Figure 5b. After the drop falling down from the wire, the flame flickered in the next few seconds. The yellow flame area then decreased sharply until it disappeared. During the second drop, the bright flame area suddenly expanded to the maximum and then decreased rapidly again until the flame was completely extinguished. The whole process from flame start to the final extinction was quite fast, and the total duration was within less than 10 s. These experimental observations suggest that the flame spread was hardly being sustained in the air at a very low ambient pressure of about 20.0 kPa.

The wire could be ignited at a higher electrical current even at 20.0 kPa, which may be due to the high heating inputs and the accompanied high pyrolysis rate for the combustible materials. Under a low oxygen mass concentration at reduced pressure, the fire spread along the wire was hampered that it was unable to provide enough heat to melt the insulation material. Under the circumstance, dripping could occur easily. On the other hand, heat supply was also reduced under the small fire. The burning was difficult to be sustained under the frequent dripping, and the flame could easily go out.

**Figure 6:**
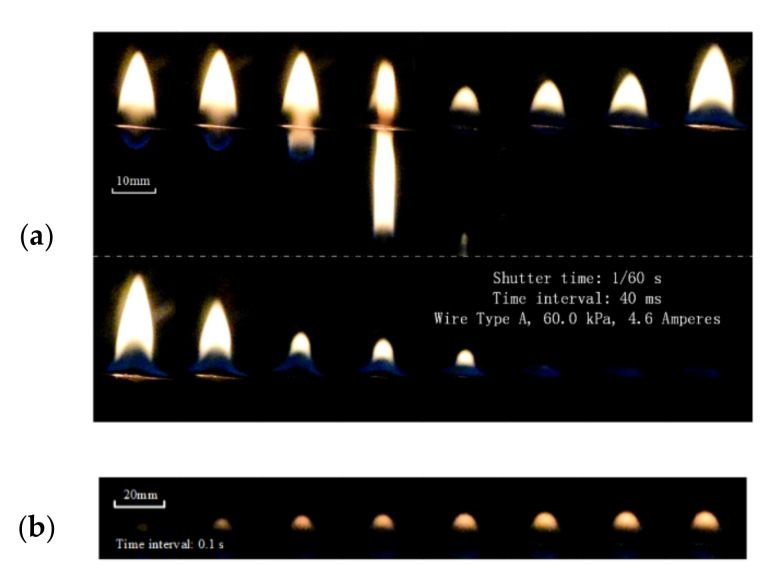
Flame disappearance in a short period of time caused by dripping at 60.0 kPa: (**a**) the bright flame flickers and disappears after a drop and (**b**) the flame shows again.

At the reduced pressure, the occurrence of dripping was not always followed by flame extinction. The bright flame could disappear for several seconds and then show again, which is a unique phenomenon for the energized wires. As shown in Figure 6, after the dripping, the flame shows a very noticeable flicker, where the bright flame height suddenly decreased, rapidly increased (the maximum even exceeded the previous peak), and then decreased until the flame disappeared. The whole process was within 0.5 s. Then after 0.8 s, a small yellow flame shows again, which expanded slowly and its brightness increased gradually.

### 3.4. Critical Dripping Point

**Figure 7:**
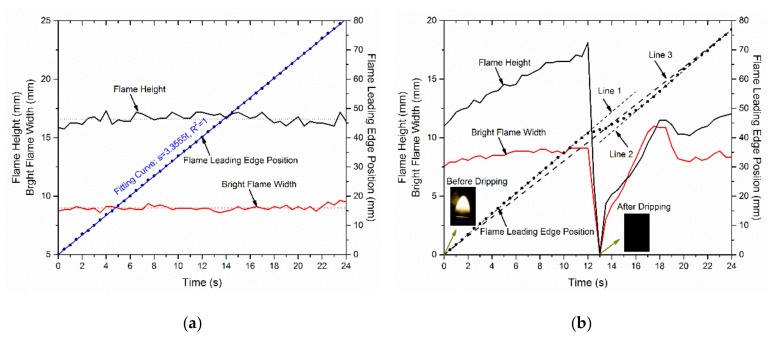
The flame height, the bright flame width and the flame front position for the wire within 24 s at 60.0 kPa. (**a**) *I* = 4.5 A and (**b**) *I* = 4.6 A.

It was known from the above analysis that the wire fire could show steady flame propagation until a certain reduced pressure and electrical current. There may be a turning point that can sustain the flame (the curve between Section 3 and Section 4 in Figure 3), which is defined as the critical dripping point in this study. This is an important criterion to determine the flame spread of wires under various conditions. To address it, several further tests close to the potential critical point were conducted under various reduced pressures. As shown in Figure 7, the wire flame spread at 60.0 kPa with: (a) no dripping occurred under I = 4.5 A and (b) dripping occurred for I = 4.6 A.

To address it, several further tests close to the potential critical point were conducted under various reduced pressures. As shown in Figure 7, the wire flame spread at 60.0 kPa with: (a) no dripping occurred under I = 4.5 A and (b) dripping occurred for I = 4.6 A. In Figure 7a, the flame height and the flame width almost keep constant at 4.5 A electrical current, without any dripping during the 24 s. Therefore, the mass of the molted insulation material could be maintained at a certain level and stable flame morphology was observed. Especially for the flame width, it was almost kept constant at 9.0 mm. The variation of the flame height was a bit higher with less than ±10% around 16.6 mm. After the calculation, the fire spread rate during the whole process was about $v_{f}$ = 3.3555 mm/s. In summary, wire under 4.5 A electrical current shows a steady fire spread without dripping.

Figure 7b shows the fire spread under an electrical current of 4.6 A. At 12.0 s, a drop occurred and the bright flame disappeared completely. Before the dripping, the flame height increased steadily from 10.9 to 18.1 mm, while the maximum growth rate was about 0.67 mm/s. Additionally, the flame width increased slightly from 7.93 to 9.12 mm, with the average growth rate was about 0.10 mm/s. Shortly after the dripping, the bright flame shows again, and then the flame spread continued. The flame height and width increased again, showing a certain periodicity. To obtain the flame spread rate, three lines marked with 1, 2 and 3, respectively, in Figure 7b, were adopted to fit the flame front position along with time. The fitting curves can be described by: Line 1, s = 3.49t, R^2^ = 0.9999; Line 2, s = 3.51t − 7.2019, R^2^ = 0.9993; Line 3, s = 3.20t, R^2^ = 0.9941.

The slope of the fitting line represents the fire spread rate. The spread rates before and after the dripping were quite similar, represented by Lines 1 and 2. However, they were quite different from the average spread rate during the whole process (Line 3). The average spread rates before and after the dropping (3.50 mm/s) were larger than that at 4.5 A electrical current (3.36 mm/s). This is consistent with the previous study [32] that the fire spread rate increases under a bigger electrical current. However, the average fire spread rate for the whole process (Line 3), including the periods before and after the dripping, was less than that at 4.5 A electrical current. This indicates that the short-term flame disappearance could stop or delay the fire spread during the dripping.

It can be concluded from the above experimental results that the wire fire spread smoothly under 4.5 A (*I*^2^*R* = 0.45 W/m) and dripping occurs under 4.6 A (*I*^2^*R* = 0.47 W/m). The critical dripping point could be determined as 4.6 A for the wire at 60.0 kPa.

Similarly, the critical dripping points under different reduced pressure could also be determined. The experimental results show that a relatively smaller current was required when the wire was under lower ambient pressure. This decreasing trend was nonlinear to ambient pressure. The whole trend could be divided into three regions: during 100–80.0 kPa, the critical dripping point decreased smoothly, while it decreased quite rapidly between 80.0 and 60.0 kPa. Once the pressure was lower than $P^{*}$ as shown in Figure 3, the flame spread was difficult to be sustained, showing no continued drops. Further research and analysis associated with $P^{*}$ will be present in the future study.

## 4. Conclusions

In this study, the dripping and flame spread behaviors of molten wire insulation (polyethylene-insulated copper core) at reduced pressures were investigated experimentally under overcurrent. Several conclusions could be addressed as follows:

1. It was known from experiments that the dripping frequency increased under lower ambient pressure, showing different trends under various electrical currents. Under a larger current, it increased more rapidly and the growth rate shows a transition from linear to power functions. The flame spread could be hardly sustained as the occurrence of dripping under reduced pressure;
2. A unique phenomenon for wire fire is observed due to the dripping behavior at reduced pressure. When the fire was spreading along the wire, the bright flame disappeared for several seconds during the dripping and then showed again. While at atmospheric pressure of 20.0 kPa or below, the wire flame was extinguished after the occurrence of the dripping;
3. When the pressure kept decreasing, the required electrical current to activate the dripping phenomenon was decreasing. The decreasing trend for the critical dripping point was nonlinear along with the pressure. The critical current changed smoothly during 100–80.0 kPa and decreased rapidly at 80.0–60.0 kPa. Additionally, the dripping phenomenon could stop or delay the flame spread, partly because of the short-term flame disappearance.

The results of this work provided a deeper understanding on the development of electrical wire fire. Additionally, it is beneficial to the design of future electrical fire protection and fire control under reduced atmospheric pressure.

## Figures and Tables

**Table 1:** Varied pressures via increasing the overload currents.

Wire Type	Core Diameter (mm)	Insulation Thickness (mm)	Rated Current (A)	Pressure (kPa)	Overload Currents (A)	Ratio of Overload to Rated
Polyethylene insulated copper wire	0.5	0.15	0.78	20–100	4–16	5–20

## Data Availability

All data, models, and code generated or used during the study appear in the submitted article.

## Nomenclature

A	cross-sectional area (m^2^)
f	dripping frequency (Hz)
I	current (A)
$I_{0}$	rated current (A)
$\dot{m}$	mass variation rate (g/s)
P	ambient pressure (kPa)
R	resistance per unit length (Ω/m)
V	volume (m^3^)
v	flame spread rate (mm/s)
ρ	density (g/mm^3^)
**Subscripts**	
c	critical dripping point
p	insulation (polyethylene)
g	gas
f	flame
lim	limitation
mlt	molten insulation
